# High-Dose-Rate Brachytherapy as Monotherapy for Low- and Intermediate-Risk Prostate Cancer. Oncological Outcomes After a Median 15-Year Follow-Up

**DOI:** 10.3389/fonc.2021.770959

**Published:** 2021-12-02

**Authors:** Manuel Behmueller, Nikolaos Tselis, Nikolaos Zamboglou, Eleni Zoga, Dimos Baltas, Claus Rödel, Georgios Chatzikonstantinou

**Affiliations:** ^1^ Department of Radiotherapy and Oncology, University Hospital Frankfurt, Goethe University Frankfurt, Frankfurt am Main, Germany; ^2^ Department of Radiotherapy, German Oncology Center, Limassol, Cyprus; ^3^ Department of Radiation Oncology, Offenbach Hospital, Offenbach am Main, Germany; ^4^ Division of Medical Physics, University Hospital Freiburg, Albert-Ludwigs University, Freiburg im Breisgau, Germany

**Keywords:** prostate cancer, HDR-brachytherapy, monotherapy, biochemical relapse free survival, toxicity

## Abstract

**Introduction:**

To evaluate the oncological outcome of high dose rate (HDR) brachytherapy (BRT) as monotherapy for clinically localised prostate cancer (PCA).

**Material and Methods:**

Between January 2002 and February 2004, 141 consecutive patients with clinically localised PCA were treated with HDR-BRT monotherapy. The cohort comprised 103 (73%) low-, 32 (22.7%) intermediate- and 6 (4.3%) high risk patients according to D’Amico classification or 104 (73.8%) low-, 24 (17.0%) intermediate favourable-, 12 (8.5%) intermediate unfavourable- and one (0.7%) very high risk patient according to National Comprehensive Cancer Network (NCCN) one. Patients received four fractions of 9.5 Gy delivered within a single implant up to a total physical dose of 38 Gy. Catheter-implantation was transrectal ultrasound-based whereas treatment planning CT-based. Thirty-three patients (23.4%) received ADT neoadjuvantly and continued concurrently with BRT. Biochemical relapse-free survival (BRFS) was defined according to the Phoenix Consensus Criteria and genitourinary (GU)/gastrointestinal (GI) toxicity evaluated using the Common Toxicity Criteria for Adverse Events version 5.0.

**Results:**

Median age at treatment and median follow-up time was 67.2 and 15.2 years, respectively. Twenty-three (16.3%) patients experienced a biochemical relapse and 5 (3.5%) developed distant metastases, with only one patient dying of PCA. The BRFS was 85.1% at 15 years and 78.7% at 18 years. The corresponding overall survival, metastases-free survival, and prostate cancer specific mortality at 15- and 18-years was 73.9%/59.1%, 98.3%/90.6%, and 100%/98.5% respectively. Late grade 3 GI and GU toxicity was 4.2% and 5.6% respectively. Erectile dysfunction grade 3 was reported by 27 (19%) patients. From the prognostic factors evaluated, tumor stage (≤T2b compared to ≥T2c) along with the risk group (low-intermediate vs. high) when using the D’Amico classification but not when the NCCN one was taken into account, correlated significantly with BRFS.

**Conclusion:**

Our long-term results confirm HDR-BRT to be a safe and effective monotherapeutic treatment modality for low- and intermediate risk PCA.

## Introduction

Prostate cancer (PCA) is the most common solid tumor in men (1). Since the introduction of serum prostate-specific antigen (PSA) testing, the incidence of PCA has substantially increased though with decreasing tendency in recent years (2). Changes in screening recommendations indicate that about 80% of patients are diagnosed with localized prostate cancer (LPC) (3). Radical treatment of LPC includes radical prostatectomy, external beam radiotherapy (EBRT), brachytherapy (BRT) and stereotactic body radiotherapy (SBRT) (4, 5). Randomized trials have supported the positive association between dose-escalated EBRT and improved clinical outcomes in patients with PCA (6, 7). However, administering doses > 74 Gy to the prostate, requires advanced imaging and planning techniques to limit dose to the adjacent critical organs. The implementation of three-dimensional (3D) conformal radiation therapy, intensity modulated radiation therapy (IMRT), and image guided radiation therapy allowed the dose escalation while reducing acute and late toxicities (8).

Compared to EBRT, BRT dosimetry offers the optimum in conformality, an unrivalled dose drop-off gradient beyond the gland, markedly sparing normal tissues and enables extreme dose intensification to the prostate (9). High-dose-rate (HDR) BRT takes advantage of the inherent sensitivity of PCA cells to hypofractionation. Recent data suggest that the a/b ratio for PCA is low compared to that of most tumors (10). Inasmuch as PCA cells maintain growth kinetics similar to those of late-responding normal tissues, there exists the potential for therapeutic gain when high doses per fraction are administered. High-dose-rate BRT as monotherapy for localised PCA was first proposed in the mid-1990s and since then several studies with mature results have proven its safety and efficacy (11). In our department, HDR-monotherapy was introduced in 2002 and subsequently developed *via* implementation of two further treatment protocols introduced in 2004 and 2008, respectively. The goal of the current study is to report the oncological outcome of our first protocol with the longest follow-up.

## Material and Methods

Since 2002, more than 1200 patients with HDR monotherapy for clinically localised PCA have been treated. During this period, three different dose-regimen were implemented, reflecting an evolution according to continuously generated radiobiological knowledge, technological advances, and patient’s feedback regarding clinical workflow. From January 2002 to February 2004, 141 patients were treated with one implant of four fractions ά 9.5 Gy. From March 2004 to January 2008, 351 patients received two implants, separated by 14 days, each of two fractions ά 9.5 Gy. Since February 2008, our ongoing HDR scheme consists of three single-fraction implants, each delivering 11.5 Gy, with an interfractional interval of 21 days.

All patients had histologically proven adenocarcinoma of the prostate and were staged according to the 6th edition of the TNM Classification of the Union for International Cancer Control. Pre-treatment staging included digital rectal examination, transrectal (TR) ultrasound (US) (TRUS) and, if clinically indicated, CT and bone scintigraphy. The D’Amico (12) classification was used to classify patients into risk groups. We additionally used retrospectively the National Comprehensive Cancer Network (NCCN) classification in order to differentiate between favourable and unfavourable intermediate risk group. Eligibility criteria were clinically organ-confined disease in the absence of severe lower urinary tract symptoms. Gland size >50 cm³ was not a contraindication, provided that there was a sufficiently broad pelvis. Patients who had previous transurethral resection of the prostate (TURP) were not excluded from treatment but assigned at six months after resection. High-risk patients who were clinically diagnosed as unsuitable for prostatectomy or dose-escalated EBRT, or who rejected prostatectomy or definitive EBRT were also assigned for HDR monotherapy at the discretion of the treating physician. Exclusion criteria were metastatic disease, previous pelvic EBRT for another malignancy and contraindication for anaesthesia. Hormonal therapy according to patients’ risk-group was prescribed by the referring urologists.

### Technique

Our technique and clinical workflow have been described in detail elsewhere (13). In short, transperineal catheter implantation was performed under TRUS-guidance in high-lithotomy position using a perineal template. For inverse preplanning, transversal US images of the prostate, bladder, urethra and anterior rectal wall were acquired in real-time using a continuous probe movement technique and 3D volumes were reconstructed based on 1.0 mm image distance. The planning target volume (PTV) was defined as the entire prostate gland without margins. Based on the acquired 3D anatomy, appropriate virtual catheter positions were generated using the intraoperative treatment planning system SWIFT/Oncentra Prostate (Oncentra Brachy, Elekta AB, Stockholm, Sweden) and dose volume histograms (DVHs) for the PTV and the organs at risk were calculated for evaluation of the anatomy-based dose optimisation. As the preplanning dosimetry parameters fulfilled our clinical protocol, TRUS-guided implantation of plastic catheters (200 mm length, 1.9 mm diameter) was performed at previously defined positions. After completion of implantation, a spiral CT scan of the pelvis (3.0 mm slice thickness) was performed, and the images was sent to the PLATO BPS (Nucletron BV, Veenendaal, Netherlands) workstation for 3D conformal post-planning. Contours of the PTV, urethra, and rectum were then delineated in all CT slices. Evaluation of implant conformity was based on dose-volume parameters for PTV coverage in compliance with organs at risk dose constraints. The D10 urethra (dose delivered to 10% of the urethra) was limited to 75% and the D10 rectum (dose delivered to 10% of the rectum) and the D10 bladder (dose delivered to 10% of the bladder) to 75% of the reference dose (Dref). Our aim was to achieve a D90 (dose delivered to 90% of the PTV) > 90% of Dref. Dose specification was given as the mean dose on the PTV surface. The reference dose was 9.5 Gy per fraction delivered four times in 48 h to a total physical dose of 38.0 Gy. The patients were immobilized during the entire treatment and received continuous i.v. infusion with meperidine (10 mg/h) for pain control. The first fraction of HDR brachytherapy was delivered on the day of implantation, second and third fraction on day 1 after implantation, with at least 6 h between the fractions, and the fourth fraction in the morning of day 2 after implantation. Before each fraction, any needle movement in the caudad-cephalad direction was controlled. After the last fraction, all flexible plastic needles were removed, and the patients were discharged home after having voided spontaneously. All implants were performed under spinal, or general anaesthesia. All treatments were performed using a 192Iridium HDR afterloading system (microSelectron–HDR, Elekta-Brachytherapy, Elekta AB, Sweden). Written informed consent was obtained from all patients. This analysis was approved by the local research ethics board.

### Statistic

All patients presented in our department at six weeks after completion of treatment and then every three months for the first two years, every six months for the next two years and annually thereafter. During the visits, apart from PSA-value recording, gastrointestinal (GI)/genitourinary (GU) toxicities were also evaluated. Gastrointestinal and GU toxicities were documented according to the National Cancer Institute Common Toxicity Criteria for Adverse Events (CTCAE), version 5.0. For the current analysis, the patient sample was deduced from our prospectively maintained database and consequently retrospectively analysed. Follow-up ended during Juli 2020 and December 2020 with all patients receiving additionally a questionnaire assessing their current PSA level and the presence as well as the grade of adverse events at that period of time. For the deceased patients the last PSA and toxicity grade documented were used and censored at that time. As late toxicities were referred side effects that began or persisted three months after treatment completion. Biochemical relapse was defined using the Phoenix criteria (sustained posttreatment PSA value > nadir +2 ng/ml) (14). Patients with an elevation of PSA followed by a drop below nadir +2 were considered having a PSA bounce. Biochemical relapse free survival (BRFS) was calculated from the date of BRT to the date of biochemical failure or initiation of androgen deprivation for presumed biochemical relapse. Metastasis-free survival (MFS) was calculated from the date of BRT to the date when distant metastases were identified. The definition of potency was noted as the ability of achieving an erection sufficient for intercourse. Using the Kaplan–Meier method, the likelihood of events was calculated and thereafter compared using the log-rank test. A two-sided p-value of < 0.05 was considered statistically significant. The Cox proportional hazards model was used for multivariate analysis. For statistical analysis, the BiAS program Version 11.10 was used.

## Results

### Oncological Outcomes

**Figure 1:**
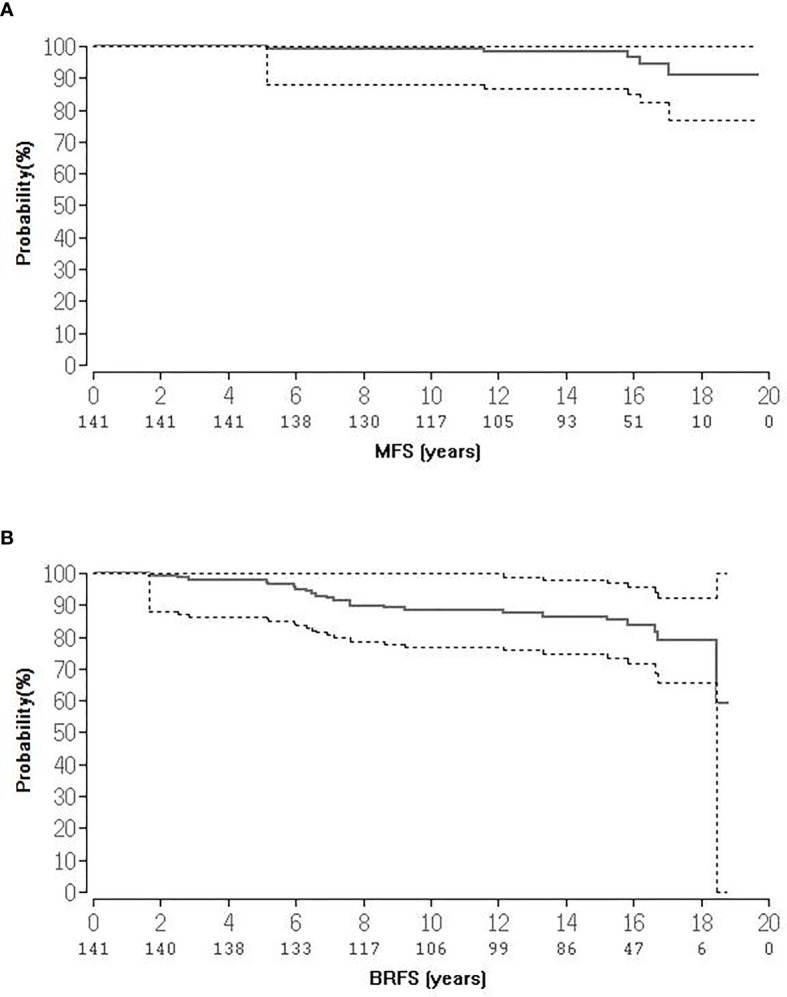
**(A, B)** Kaplan-Meier survival curves of BRFS and DMFS.

**Figure 2:**
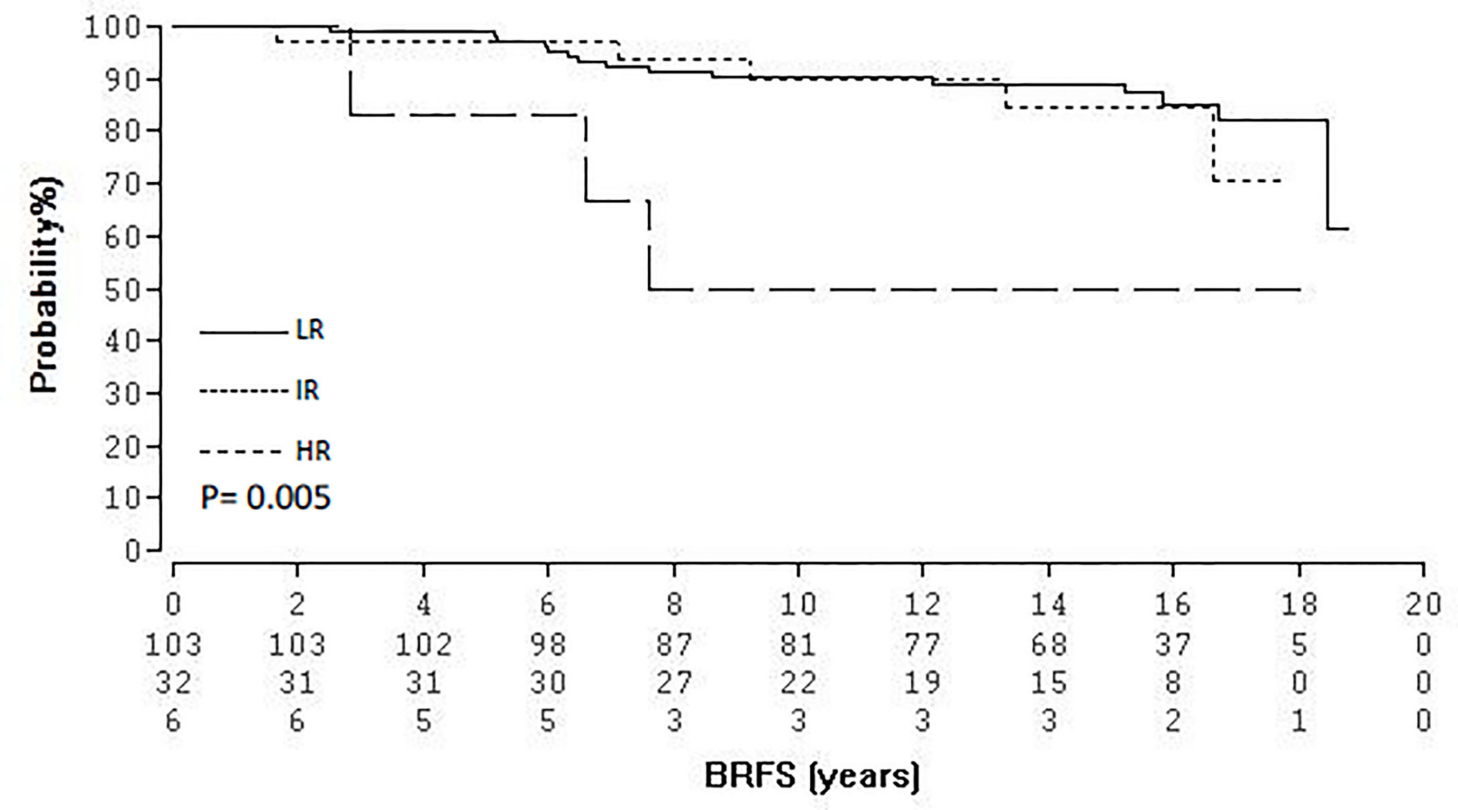
Kaplan-Meier survival curves of BRFS according to the D’Amico classification.

**Figure 3:**
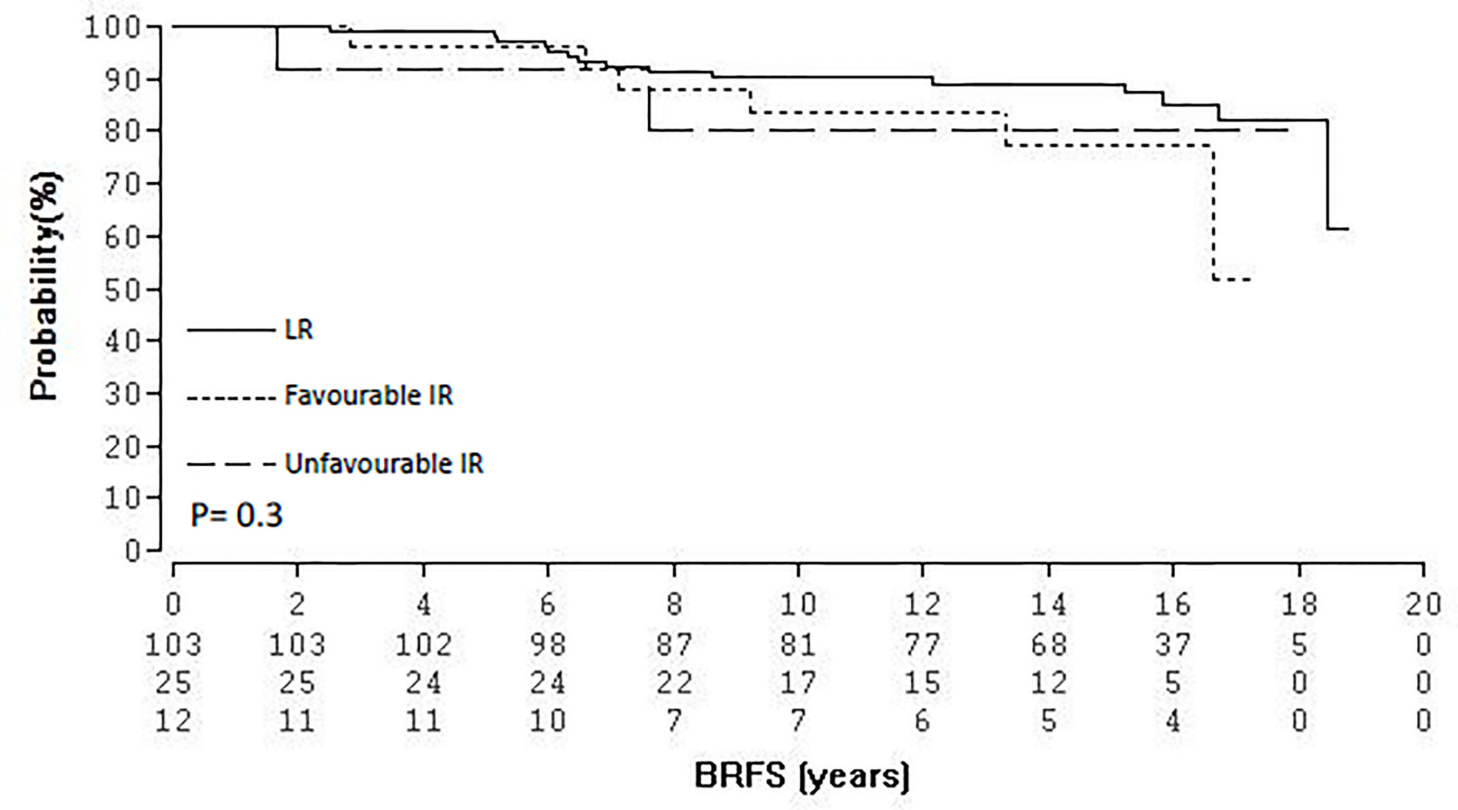
Kaplan-Meier survival curves of BRFS according to the NCCN classification.

At the time of follow-up cut-off (December 2020) 90 (63.8%) patients were still alive and 45 (31.9%) have died. Six (4.2%) patients were lost to follow up since the previous analysis (15). Of the 45 deceased patients 44 patients died of causes other than PCA. Tumor and patient’s characteristics are shown in **Table 1**. Thirty-three patients (23.4%) received androgen deprivation therapy prescribed neoadjuvantly and continued concurrently with BRT. Median follow-up for the entire cohort was 15.2 years (range, 2.5-18.7) and 15.9 years (range, 6.4-18.7) for patients alive. Twenty-three (16.3%) patients experienced a biochemical relapse and 5 (3.5%) developed distant metastases, with only one patient dying so far as of PCA. The BRFS was 85.1% at 15 years and 78.7% at 18 years. The corresponding overall survival (OS), MFS and prostate cancer specific mortality (PCSM) at 15- and 18-years were 73.9%/59.1%, 98.3%/90.6%, and 100%/98.5% respectively. **Figures 1A, B** shows the Kaplan-Meier curves for BRFS and MFS. With regard to D’Amico risk group stratification, actuarial BRFS at 15- and 18-years was 88.9/81.9%, 84.6%/70.5%, and 50%/50% for low-, intermediate- and high-risk patients, respectively, proven to be statistically significant for low-intermediate to high risk patients (p = 0.005, **Figure 2**). With reference to NCCN risk group stratification and excluding the one patient categorised as very high risk, BRFS at 15- and 17-years was 87.3%/81.9%, 77.4%/51,6%, and 82%/82% for low-, intermediate-favourable and intermediate unfavourable risk patients, respectively, proven to be statistically non-significant (p = 0.3), **Figure 3**. Based on pre-treatment PSA, BRFS at 15- and 18- years was 87.1%/78.8 vs. 77.7%/77.7% for iPSA ≤10 vs. > 10 ng/ml, respectively, without showing statistical significance (p = 0.7). According to Gleason score (GS), BRFS at 15-years was 95.4%, 85.4 and 50% for patients with GS ≤6, 7a and ≥7b, respectively, also not showing statistical significance (p = 0.5). With regard to clinical tumour stage (cT), BRFS at 15- and 18-years was 88.7%/80.8%%, 80%/80% and 50/50% for patients with clinical stages ≤T2a, T2b and ≥T2c, respectively, being statistically significant for ≤T2a and T2b compared to ≥T2c (p = 0.005). In addition, the role of androgen deprivation therapy as well as the one of median age relative to BRFS were also analysed and found not to be significantly correlated. The same held true if the univariate analysis was undertaken according to the median PSA (≤ 6.5 ng/ml vs. 6.5).

**Table 1:** Patients and tumor characteristics.

Characteristic	n = 141 (%)
**Median volume at implant (cm³) (range)**	**40 (20 – 90)**
**Median age at treatment (years) (range)**	67.2 (46 - 79.9)
**Stage**	
T1c	58 (41.1)
T2a	66 (46.8)
T2b	11 (7.8)
T2c	5 (3.5)
T3a	1 (0.7)
**Gleason Score**	
≤6	114 (80.8)
7	26 (18.4)
≥8	1 (0.7)
**Median pre-treatment PSA (ng/ml) (range)**	6.5 (2.1 – 58)
≤10	133 (94.3)
10.1-20	7 (5.0)
>20	1 (0.7)
**D’Amico risk classification**	
low	103 (73)
intermediate	32 (22.7)
high	6 (4.3)
**NCCN risk classification**	
low	104 (73.8)
intermediate favourable	24 (17.0)
intermediate unfavourable	12 (8.5)
very high	1 (0.7)

The median age and iPSA of the 23 (16.3%) patients that recurred biochemically was 67.1 years (range, 50.2-77.9) and 6.7 ng/ml (range, 4.0-11.3). Twenty patients (86.9%) had a GS ≤6 and three (13.1%) had a GS 7, whereas 18 (78.2%), 3 (13.1%), and 2 (8.7%) had a clinical T stage ≤T2a, T2b and ≥T2c, respectively. According to the D’Amico classification 15 (65.2%) patients were classified as having low-, 5 (21.7%) as intermediate- and 3 (14.2%) as high-risk PCA. For the 5 (3.5%) patients that developed metastases median age and iPSA was 65.4 years (range, 53.6-70.6) and 6.1 ng/ml (range, 5.8-9.3), respectively. Of these patients, 3 (60%) presented with a clinical T stage T2a, 2 (40%) with T2b, 4 (80%) had a GS ≤6 and one (20%) a GS 7, respectively. Three (60%) had low- and two (40%) intermediate- risk PCA.

### Toxicity

In contrast to our previous report, toxicity is reported according to the CTCAE version 5.0. **Table 2** shows the results for acute toxicity. There was only one case of GI toxicity grade ≥ 2, whereas GU toxicity of grade 2 and 3 was documented in 15.6% and 9.2% of patients, respectively. **Table 3** shows the corresponding results for late GU and GI adverse events. Two patients (1.4%) developed late grade 2 GI toxicity. Six (4.2%) patients suffered from grade 3 GI toxicity, including two (1.4%) patients with rectal necrosis requiring endoscopic restoration of bowel continuity and permanent colostomy, respectively and three (2.1%) patients with rectal mucositis who underwent laser coagulation for rectal bleeding. Overall, 8 (5.6%) patients suffered grade 3 GU late toxicity among which 5 (3.5%) patients who required urethrotomy for a urethral stricture and one (0.7%) requiring permanent urostomy for a grade 3 urinary incontinence. Erectile dysfunction grade 3, defined as consistent inability to sustain an erection sufficient for sexual intercourse despite impotence agents, was reported by 7 (4.9%) patients prior to treatment, whereas grade 1 and 2 by 28 (19.8%) and 16 (11.3%), respectively. After BRT an increase of all grade erectile dysfunction was noted with grade 1, 2, 3 reported by 50 (35.4%), 35 (24.8%) and 27 (19%) patients, respectively. When considering the 92 patients alive the corresponding grade 1, 2 and 3 rates was 28.2%, 26% and 11.9% respectively.

**Table 2:** Acute toxicity results.

Grade	No. of occurrences (%)	
	Gastrointestinal	Genitourinary
1	26 (18.4%)	66 (46.8%)
2	0	22 (15.6%)
3	1 (0.7%)	13 (9.2%)
4	0	0
5	0	0

**Table 3:** Late toxicity results.

Toxicity	No. of occurrences (%)				
	Grade				
	1	2	3	4	5
**Genitourinary**					
Frequency	37 (26.2)	3 (2.1)	–	–	–
Urgency	11 (7.8)	13 (9.2)	–	–	–
Dysuria	9 (6.3)	1 (0.7)	1 (0.7)	0	0
Incontinence	7 (4.9)	11 (7.8)	2 (1.4)	–	–
Retention/stricture	22 (15.6)	9 (6.3)	5 (3.5)	–	0
Erectile Dysfunction	50 (35.4)	35 (24.8)	27 (19.1)	–	–
**Gastrointestinal**					
Pain	2 (1.4)	1 (0.7)	1 (0.7)	–	–
Mucositis	0	1 (0.7)	3 (2.1)	0	0
Rectal necrosis	–	–	2 (1.4)	0	0
Diarrhea	1 (0.7)	0	0	0	0

There was no correlation found between late grade 3 genitourinary or gastrointestinal toxicity and the dosimetric parameters used for the treatment planning, namely D10 < 75% for rectum, bladder, and urethra.

## Discussion

Our retrospective analysis shows that HDR-BRT as monotherapy yields excellent long-term BRFS for low- and intermediate risk PCA-patients with relative low grade 3 toxicity rates. To the best of our knowledge, this is the first report regarding HDR-BRT as monotherapy with a median follow-up of 15 years.

Since our previous publication (15) 13 additional biochemical relapses have been documented for a median BRFS of 85.1% at 15 years and 78.7% at 18 years for the entire cohort and 88.9/81.9%, 84.6%/70.5% for low and intermediate risk patients respectively. In the publication with so far, the longest follow-up (16), Yoshioka et al. treated 79 intermediate- and 111 high-risk PCA-patients with HDR-BRT alone with different dose schedules, namely 48 Gy/8 fractions, 54 Gy/9 fractions, or 45.5 Gy/7 fractions over 4 to 5 days. After median 92 months, the BRFS was 83% for all patients and 91% and 77% for the intermediate- and high risk ones, respectively, corroborating our results and highlighting the efficacy of HDR-BRT as monotherapy even for high-risk patients.

Our long-term BRFS results compare favourably with other radiotherapy modalities used in the treatment of localised PCA. Sylvester et al. (17) reported on their outcomes following low-dose-rate BRT as monotherapy with a prescription I^125^ dose of 160 Gy. Their cohort comprised 128 low and 36 intermediate risk patients according to the D’Amico classification. With a median follow-up of 11.7 years and 15.4 years for the biochemically free of disease patients, 15-year BRFS was 80.4% for the entire cohort and 85.9% and 79.9% for low- and intermediate risk patients, respectively. Weg et al. (18) analysed retrospectively their results of dose-escalated IMRT for a total dose of ≥ 81 Gy. The study population consisted of 95 low-, 140 intermediate- and 66 high-risk patients according to the NCCN classification. The median follow-up was 12 years, and for the patients alive 13.8 years. The 15-year BRFS was 76% for the low- and 65% for the intermediate risk group. With regard to SBRT, the study with the median longest-follow-up (although not extending 10 years) is the one by Katz (19), in which 230 low-risk PCA-patients were treated with Cybeknife in 5 fractions over consecutive days for a total dose of 35 (36.25) Gy. After median 9 years, the 10-year BRFS was 93.7%.

Our toxicity profile is line with the current literature presenting long-term side effects. There was no acute or late grade 4 toxicity documented, for a late grade 3 GI and GU toxicity of 4.2% and 5.6% respectively. In a recently published review regarding the toxicity of HDR-BRT as monotherapy for the treatment of localised PCA (20), late grade 3 GU and GI toxicity was in the range of 0-6% and 0-2%, respectively, with the latter one being slightly lower than in our study. One possible explanation for our slightly higher late grade 3 GI toxicity as well as for the relatively high, though still in the range of 6%, grade 3 GU toxicity is the transrectal approach during catheter placement as well as the CT-based treatment planning, as the rate of late grade 3 GI and GU toxicity accounts for 0% and 0.6%, respectively in our current evolved treatment protocol being fully transperineal- and TRUS-based (21). The side effects of LDR-BRT are summarised in the review by Helou et al. (22). With regard to GU toxicity, late grade 3 occurs in < 10%, whereas there are also some very rare cases of grade 4 (<1%). Late grade ≥ 3 GI toxicity is very uncommon with occurrence rates of < 2%. Regarding dose escalated IMRT, in the study by Weg et al. (18) late grade 3, and 4 GU toxicity was observed in 2.0%, and 0.3% of the patients, respectively, whereas late grade 2 and 3 GI toxicity was noted in 1.0% of the patients each. Low rates of late grade 2 GI toxicity of 4% and no grade 3 was observed in the SBRT study by Katz (19), whereas late GU toxicity occurred in 9% (grade 2) and 3% (grade 3) of the patients.

Since our previous publication (15) there was a light further decline of the erectile function, leaving overall 81% of the patients having an erection adequate for intercourse with or without medical aid whereas 19% suffered an erectile dysfunction grade 3. These results are comparable with the published literature. At that for example, in the LDR-BRT series from Cosset et al. (23) treating 675 low and intermediate-risk patients and after median 11 years follow-up, 61% of the patients retained an erectile function sufficient for intercourse. In the SBRT series from Katz (19) 56% of the patients who were potent prior to SBRT, remained also potent at last follow-up. Among the 384 potent patients receiving dose-escalated IMRT up to 86.4 Gy in the study by Cahlon et al. (24) 115 (30%) became impotent, with 80% of them having received ADT.

The evaluation of prognostic factors and its correlation to BRFS in our analysis revealed tumor stage (≤T2b compared to ≥T2c) as being statistically significant along with the risk group (low-intermediate vs. high) when using the D’Amico classification but not when the NCCN one was taken into account. Median iPSA and the risk group (low-intermediate vs. high) classification correlated significantly with BRFS in the study by Soatti et al. (25), whereas iPSA with a cut off of 10 ng/ml and age were of statistical significance in the study by Johansson et al. (26), both treating low-, intermediate-risk PCA-patients with HDR-BRT as monotherapy. In the univariate analysis of 1100 low- and intermediate-risk PCA-patients treated with LDR-BRT, Crook et al. (27) found risk group, GS and PSA level to be predictive of BRFS, with risk group and GS retaining significance also in the multivariate analysis. In the study by Weg et al. (18), on multivariate analysis all parameters evaluated apart from age, namely pre-treatment PSA, GS, tumor stage as well as the use of ADT predicted for BRFS. The divergence regarding predictive factors among the aforementioned studies are probably attributed to differences in the total number and tumor parameters of the included patients despite all belonging to the low or intermediate risk group.

Our study has some limitations. Firstly, its retrospective nature is associated with intrinsic bias. Furthermore, of the 45 deceased patients 44 patients died of causes other than PCA, thus limiting the follow-up and potentially confounding the occurrence of further biochemical recurrences, although this is well to expect when treating PCA-patients with a median age of 67 years. As our patients were treated between 2002-2004, staging did not include MRI, which was not a standard staging examination at that time according to the german clinical practice guidelines and as so significant PCA might have been missed and patients might have been understaged. Despite these limitations, however, we believe that our analysis is a contribution to the literature because it provides the longest to date follow-up data on the treatment of low- and intermediate-risk PCA with HDR-BRT monotherapy.

## Conclusion

With a median 15-year follow-up our single-institution study demonstrates that HDR-BRT as monotherapy is a safe and effective alternative to EBRT for the treatment of patients with low- and intermediate-risk PCA. Long-term results of prospective randomized trials comparing HDR-BRT with dose-escalated hypofractionated EBRT and SBRT are warranted to define the optimal hypofractionated radiotherapy modality.

## Data Availability Statement

The raw data supporting the conclusions of this article will be made available by the authors, without undue reservation.

## Ethics Statement

The studies involving human participants were reviewed and approved by Ethics committee of the University Hospital of Goethe University Frankfurt. Written informed consent for participation was not required for this study in accordance with the national legislation and the institutional requirements.

## Author Contributions

Conception and design: GC, NT, and MB. Provision of study materials or patients: GC, MB, EZ, and NT. Collection and assembly of data: GC, EZ, MB, and NT. Data analysis and interpretation: All authors. All authors contributed to the article and approved the submitted version.

## Conflict of Interest

The authors declare that the research was conducted in the absence of any commercial or financial relationships that could be construed as a potential conflict of interest.

## Publisher’s Note

All claims expressed in this article are solely those of the authors and do not necessarily represent those of their affiliated organizations, or those of the publisher, the editors and the reviewers. Any product that may be evaluated in this article, or claim that may be made by its manufacturer, is not guaranteed or endorsed by the publisher.
